# The role of coronary artery calcium scoring in the prediction of coronary artery disease based on non-contrast non-cardiac chest CT scans in airline pilots

**DOI:** 10.3389/fcvm.2025.1511358

**Published:** 2025-04-24

**Authors:** Lin Zhang, Li Li Liu, Zheng Bin Zhu, Yan Xu, Kai Chen, Qing Qing Duan, Yu Kai Li, Jie Gao, Meng Song, Qiu Yu Shen, Shao Jie Zhu, Qing Qing Jin, Jian Ping Wen, Shuo Feng, Ying Lu, Run Du, Bin Ren, Rui Yan Zhang

**Affiliations:** ^1^ CAAC East China Aviation Personnel Medical Appraisal Center, Civil Aviation Shanghai Hospital, Shanghai, China; ^2^ Department of Cardiovascular Medicine, Rui Jin Hospital, Jiao Tong University School of Medicine, Shanghai, China; ^3^ Department of Aviation Health, China Eastern Airlines Co., Ltd, Shanghai, China

**Keywords:** non-contrast non-gated computed tomography (NCCT), coronary computed tomography angiography (CCTA), coronary artery calcium score (CACS), Agatston, airline pilots

## Abstract

**Background:**

The aim of the present study was to explore the value of coronary artery calcium score (CACS) using non-gated, non-contrast chest computed tomography (NCCT) to predict coronary artery disease (CAD) in airline pilots.

**Methods:**

Pilots with coronary calcification found on NCCT were consecutively enrolled into this study. All received a coronary computed tomography angiography (CCTA) examination. The coronary artery calcium score (CACS) was evaluated on NCCT using the Agatston method. CCTA images were analyzed using a semi-automated software. Coronary Artery Disease Reporting and Data System (CAD-RADS) scoring categorized coronary stenosis.

**Results:**

A total of 217 male pilots were included, of which 49 were diagnosed with significant CAD (CAD-RADS category 3 or higher). Pilots with significant CAD had much higher CACS (324.28 ± 389.02 vs. 39.16 ± 68.88; *p* < 0.001). Plaque volumetric measurements showed that total plaque volume (1,103.50 ± 285.51 mm^3^ vs. 913.18 ± 277.45 mm^3^; *p* < 0.001) and calcified plaque volume (149.77 ± 160.71 mm^3^ vs. 36.42 ± 26.86 mm^3^; *p* < 0.001) were more pronounced in individuals in the significant CAD group than those in the non-significant CAD group. A multivariate analysis demonstrated that CACS (odds ratio 1.01; 95% confidence interval 1.005–1.014; *p* < 0.001) was the only independent risk factor of significant CAD but traditional cardiovascular risk factors, pre-existing medication regimens, or prolonged flight duration were not. CACS positively correlated with total plaque volume (*r* = 0.156; *p* = 0.027) and calcified plaque volume (*r* = 0.434; *p* < 0.001). Receiver operating characteristic curve analysis showed the area under the curve for the CACS in diagnosing significant CAD was 0.891 (*p* < 0.001).

**Conclusions:**

CACS assessed using NCCT was significantly associated with CAD-RADS category 3 or higher, as confirmed by CCTA, which indicates that it may serve as a robust predictor for diagnosing significant CAD among airline pilots.

## Introduction

Cardiovascular diseases (CVDs) are the leading cause of deaths worldwide and are projected to still be the single leading cause of death globally by 2030 (1). The majority of these CVD deaths are attributable to coronary artery disease (CAD), of which the most serious and devastating clinical manifestation was myocardial infarction (MI). At least 25% of patients who develop MI and sudden death have no previous warning symptoms (2). Therefore, the identification of asymptomatic individuals who are at greater risk of MI is extremely significant for the implementation of primary preventive strategies. Commercial airline pilots, who work in the aviation industry, are responsible for the safety and efficient transportation of passengers. The health of pilots is of great importance and closely related to public security. The sudden impairment of pilots’ health could lead to catastrophic consequences for many others (3, 4). Therefore, early detection of severe CAD in pilots without a prior diagnosis is crucial to preventing MI and sudden death.

The severity of coronary stenosis is classified according to the Coronary Artery Disease Reporting and Data System (CAD-RADS), which ranges from 0 to 5. Categories 3, 4, and 5 are considered indicative of significant CAD (5). Coronary artery calcium score (CACS), a non-invasive quantitative assessment using non-contrast chest computed tomography (NCCT), has been proven to be associated with the presence and extent of atherosclerotic plaque (6, 7). Furthermore, there was a strong association between CACS and cardiovascular outcomes in asymptomatic patients (8, 9).

Although multiple studies with CACS have been published, most were confined to general cohorts. The aim of the present study was to assess the role of CACS measured by NCCT in identifying significant CAD within a cohort of airline pilots in China.

## Methods

### Study population

**Figure 1:**
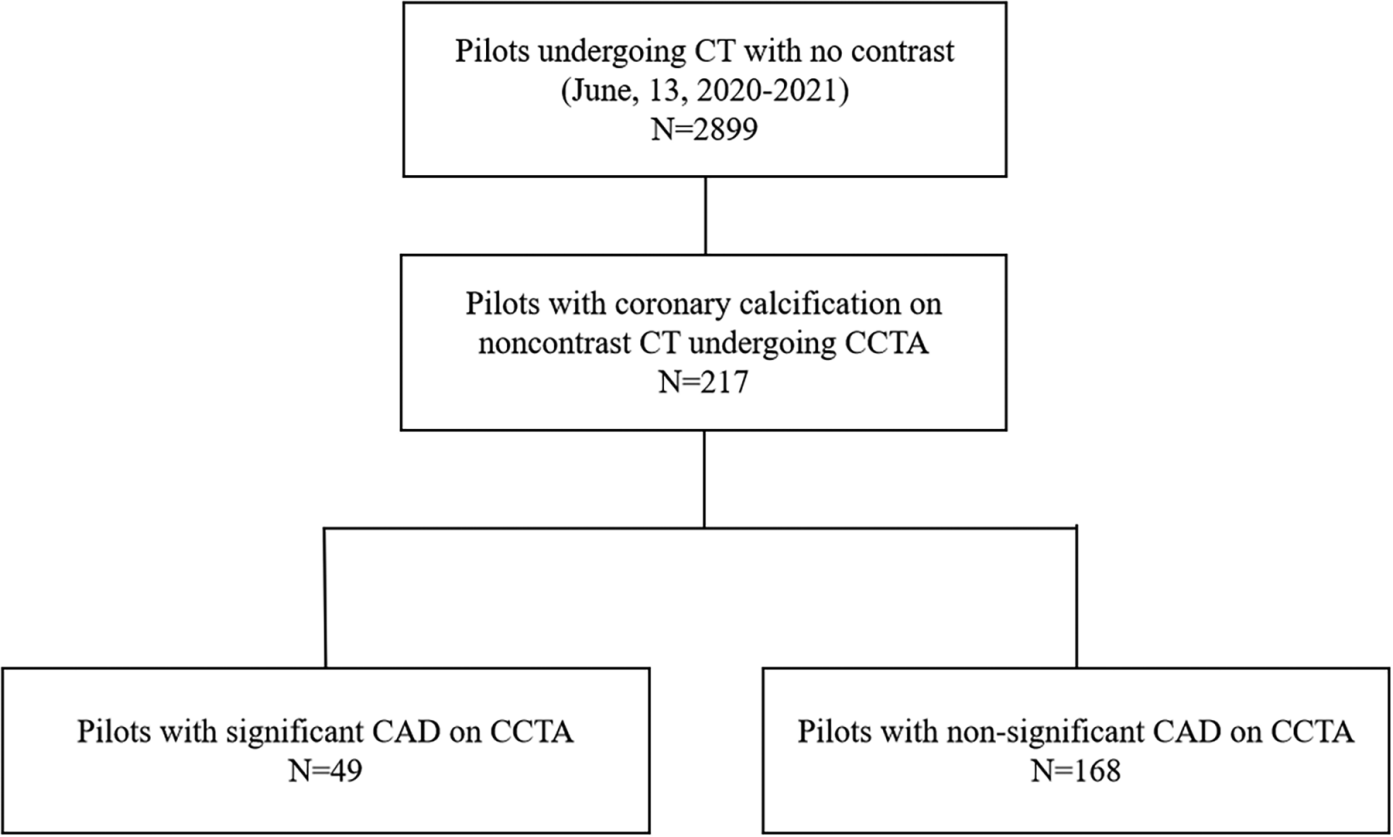
Flow chart of patient inclusion.

A total of 2,899 male pilots aged 21–60 years underwent regular check-ups in CAAC East China Aviation Personnel Medical Appraisal Center between January 2020 and December 2022. NCCT was performed for all the pilots. Of them, 217 pilots without a history of CAD who were found via NCCT to have coronary calcification received a coronary computed tomography angiography (CCTA) examination and were enrolled into this study (Figure 1). The diagnosis of type 2 diabetes mellitus (T2DM) was made according to the American Diabetes Association criteria [symptoms of diabetes included casual plasma glucose concentration ≥200 mg/dl (11.1 mmol/L) or fasting blood glucose (FBG) ≥ 126 mg/dl (7.0 mmol/L), 2 h postprandial glucose (2 h PG) ≥ 200 mg/dl (11.1 mmol/L) during an oral glucose tolerance test, glycated hemoglobin (HbA1c) ≥6.5% (48 mmol/mol), and currently or previously treated with insulin and/or oral hypoglycemic agents]. Hyperlipidemia was diagnosed in patients with total cholesterol levels of 200 mg/dl or higher and/or low-density lipoprotein (LDL) cholesterol levels of 130 mg/dl or higher and/or triglyceride values of 150 mg/dl or higher and/or high-density lipoprotein (HDL) cholesterol levels below 40 mg/dl or in patients with a present history of anti-hyperlipidemia drug use. The study was approved by the ethics committee of Ruijin Hospital.

### Image acquisition and analysis

Non-gated NCCT was performed for all the pilots. All CT scans were performed with a second-generation 128-slice dual-source CT (DSCT) scanner (SOMATOM Deﬁnition Flash; Siemens Healthcare, Erlangen, Germany) with a gantry rotation time of 280 ms. Initially, non-gated NCCT scans were performed with a pitch of 1.2. The patients underwent scanning in the supine position while holding their breath at the end of each inspiration. The CT images of the thorax from the lung apex to the adrenal glands were acquired at a voltage of 120 kVp and a reference tube current of 180 mAs. The acquisition was performed at a slice thickness of 5 mm, followed by reconstruction with a slice thickness of 1 mm and a slice interval of 0.7 mm. The axial images were transferred to a workstation for CAC scoring according to the Agatston method using a commercially available software (Circle Cardiovascular Imaging: cvi42 version 6.1, Calgary, Canada). Total Agatston CAC score was calculated as the sum of the individual lesion scores in all coronary arteries and stratified into three groups: mild (1–100), moderate (101–300), and severe (>300) (10–12).

CCTA examinations were performed separately using a second-generation 128-slice DSCT scanner (SOMATOM Deﬁnition Flash; Siemens Healthcare, Erlangen, Germany) with a tube voltage of 100 kVp and a reference tube current of 310 mAs in a dose auto-modulation system. Scanning and image reconstruction procedures were performed in accordance with Society of Cardiovascular Computed Tomography (SCCT) guidelines (13). The scanning area, starting from the level of the carina, extends to the diaphragm surface of the heart. The spiral acquisition with prospective or retrospective electrocardiography (ECG) gating scan was selected based on the patients’ heart rate. The field of view was set according to the size of individual patients, resulting in a mean pixel size of 0.36 ± 0.04 mm (range 0.30–0.48 mm). Image reconstruction was performed with a slice thickness of 0.75 mm, reconstruction interval of 0.5 mm, and a SAFIRE reconstruction algorithm (I26f) at the best diastole or best systole phase. For the contrast-enhanced scan, contrast medium (Iopromide, Ultravist 370 mg iodine/ml, Bayer Healthcare, Berlin, Germany) was injected into the cubital vein at a rate of 4–5 ml/s with a dose of 50–70 ml dependent on the patient’s size, followed by 50 ml of normal saline at the same injection rate.

**Figure 2:**
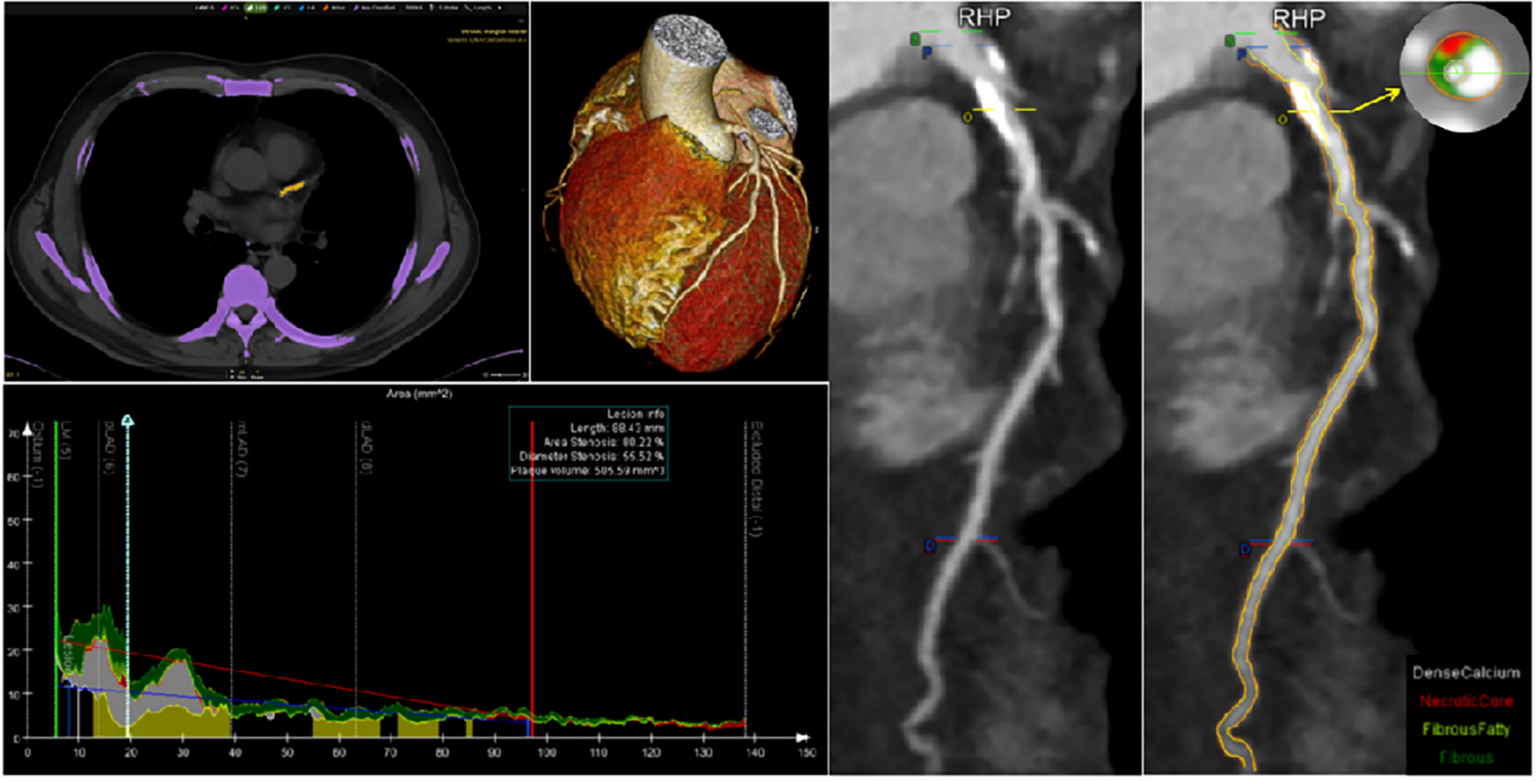
Paradigmatic example of CAC by non-contrast CT, severity of stenosis, and plaque volume analysis by CCTA in left coronary artery. CAC, coronary artery calcium; CCTA, coronary computed tomography angiography; CT, computed tomography.

Blinded image analysis and standardized measurements were performed by an independent experienced reader with over 5 years of experience using semi-automated plaque analysis software (QAngioCT Research Edition version 2.1.9.1; Medis Medical Imaging Systems, Leiden, Netherlands). After the extraction of the coronary tree from the raw data, the inner vessel lumen and the outer vessel wall from the ostium to the distal end of each artery were automatically traced with appropriate manual correction. For each segment of the 18-segment SCCT model with a diameter >2 mm, quantitative analysis was performed on every 1-mm cross-section to measure vessel length, volume, plaque volume (PV), mean plaque burden (PB), and plaque composition using pre-defined Hounsfield unit (HU) thresholds: necrotic core (NC) (−30 to 30 HU), fibro-fatty (30–130 HU), fibrous (131–350 HU), and calcified plaque (>350 HU). For each lesion, measurements were performed of minimum luminal diameter, minimum luminal area, percent diameter stenosis (%DS), area stenosis, cross-sectional PB, and mean PB. Segment-based PVs and lesion-based measurements were summarized to the patient level (Figure 2).

**Figure 3:**
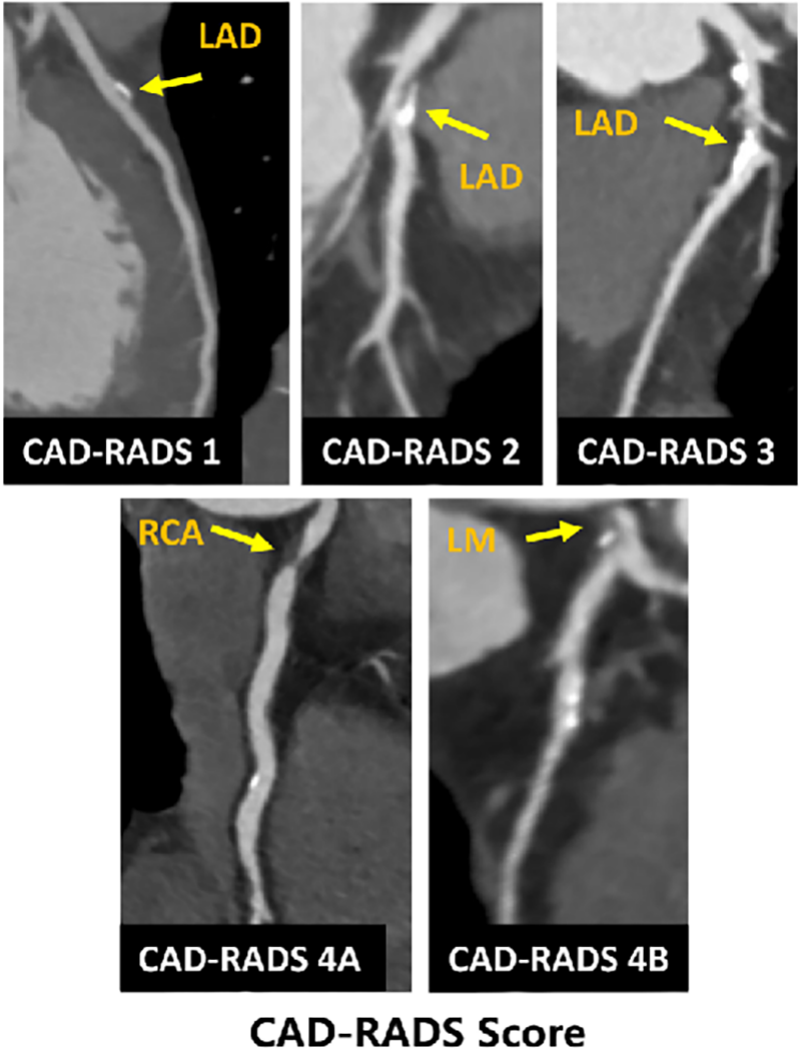
Paradigmatic example of CAD-RADS score classifications based on the study population.

CAD-RADS, which has been proven to provide more specific information regarding the location and extent of coronary plaque and stenosis, was used to evaluate the severity of CAD (5). According to the highest grade of coronary stenosis detected in any vessel, the CAD-RADS score classification is divided as follows: (1) CAD-RADS 0: 0%, (2) CAD-RADS 1: 1%–24%, (3) CAD-RADS 2: 25%–49%, (4) CAD-RADS 3: 50%–69%, (5) CAD-RADS 4A: 70%–99%, (6) CAD-RADS 4B: left main >50% or three-vessel obstruction, and (7) CAD-RADS 5: 100%. CAD-RADS categories 3, 4, and 5 were defined as significant CAD (Figure 3).

### Statistical analysis

Statistical analysis was performed using the SPSS statistical package, version 26 (IBM Corp., USA). Continuous variables were presented as mean ± SD and compared using a one-way ANOVA with a *post-hoc* analysis in two-group comparisons using Fisher’s protected least significant difference (PLSD) or Dunnett's T3 tests. Categorical variables were presented as frequencies and compared using a chi-square test or Fisher's exact test. The strength of association between two continuous variables was evaluated with partial correlation analysis by adjusting for clinically relevant covariates including age, body mass index (BMI), risk factors, and pre-existing medication regimens. To identify independent predictors of significant CAD, logistic regression was used. Those variables associated with significant CAD were entered into the multivariate model. All tests of significance were two-tailed and *p* < 0.05 was considered significant.

## Results

A total of 217 pilots with coronary calcium received a CCTA examination. Of them, 49 (22.6%) pilots were diagnosed with significant CAD (CAD-RADS category 3 or higher) and 168 pilots were diagnosed with non-significant CAD.

### Baseline characteristics

Table 1 shows the baseline characteristics of patients. The mean age was 50.62 ± 8.37 years. The median total flight time was 17,000 h. Compared with pilots with non-significant CAD, pilots with significant CAD were older (53.16 ± 5.31 years vs. 49.29 ± 10.47 years; *p* < 0.001). There were more smokers in the significant CAD group than in the non-significant CAD group (79.6% vs. 60.7%; *p* = 0.015). No significant differences were observed between the two groups regarding the proportion of hypertension, diabetes mellitus (DM), and dyslipidemia. The proportion of medications, including the intake of statins, antihypertensives, and hypoglycemic agents, was similar between the two groups. Pilots with and without significant CAD had similar total flight times and flight time/year (all *p* > 0.05).

**Table 1:** Baseline characteristics.

Parameters	Significant CAD	Non-significant CAD	*p*-value
	*N* = 49	*N* = 168	
Age (years)	53.16 ± 5.31	49.29 ± 10.47	<0.001
Height (cm)	1.75 ± 0.05	1.75 ± 0.05	0.476
Weight (kg)	74.24 ± 6.60	75.27 ± 9.84	0.491
BMI (kg/m^2^)	24.35 ± 1.63	24.50 ± 2.60	0.625
Risk factors, *n* (%)			
Hypertension	12 (24.5)	36 (21.4)	0.650
Diabetes mellitus	9 (18.4)	28 (16.7)	0.781
Dyslipidemia	24 (49.0)	65 (38.7)	0.198
Smoking	39 (79.6)	102 (60.7)	0.015
Medications, *n* (%)			
Beta-blockers	3 (6.1)	6 (3.6)	0.431
Calcium antagonists	5 (10.2)	15 (8.9)	0.782
ACEI/ARB	6 (12.2)	20 (11.9)	0.949
Hypoglycemic agents	7 (14.2)	22 (13.1)	0.829
Statins	2 (4.1)	5 (3.0)	0.657
Total flight time (h)	18,795.45 ± 5,890.44	16,918.00 ± 6,191.78	0.056
Flight time/year (h)	547.20 ± 176.92	575.60 ± 190.48	0.352

ACEI, angiotensin-converting enzyme inhibitors; ARB, angiotensin II receptor blockers; BMI, body mass index.

### NCCT and CCTA findings

Compared to pilots with non-significant CAD, pilots with significant CAD had a much higher Agatston CAC score (324.28 ± 389.02 vs. 39.16 ± 68.88; *p* < 0.001). The proportion of moderate (20.4% vs. 6.0%) and severe (32.7% vs. 1.2%) CACS was higher in the significant CAD group than in the non-significant CAD group (*p* < 0.001).

**Figure 4:**
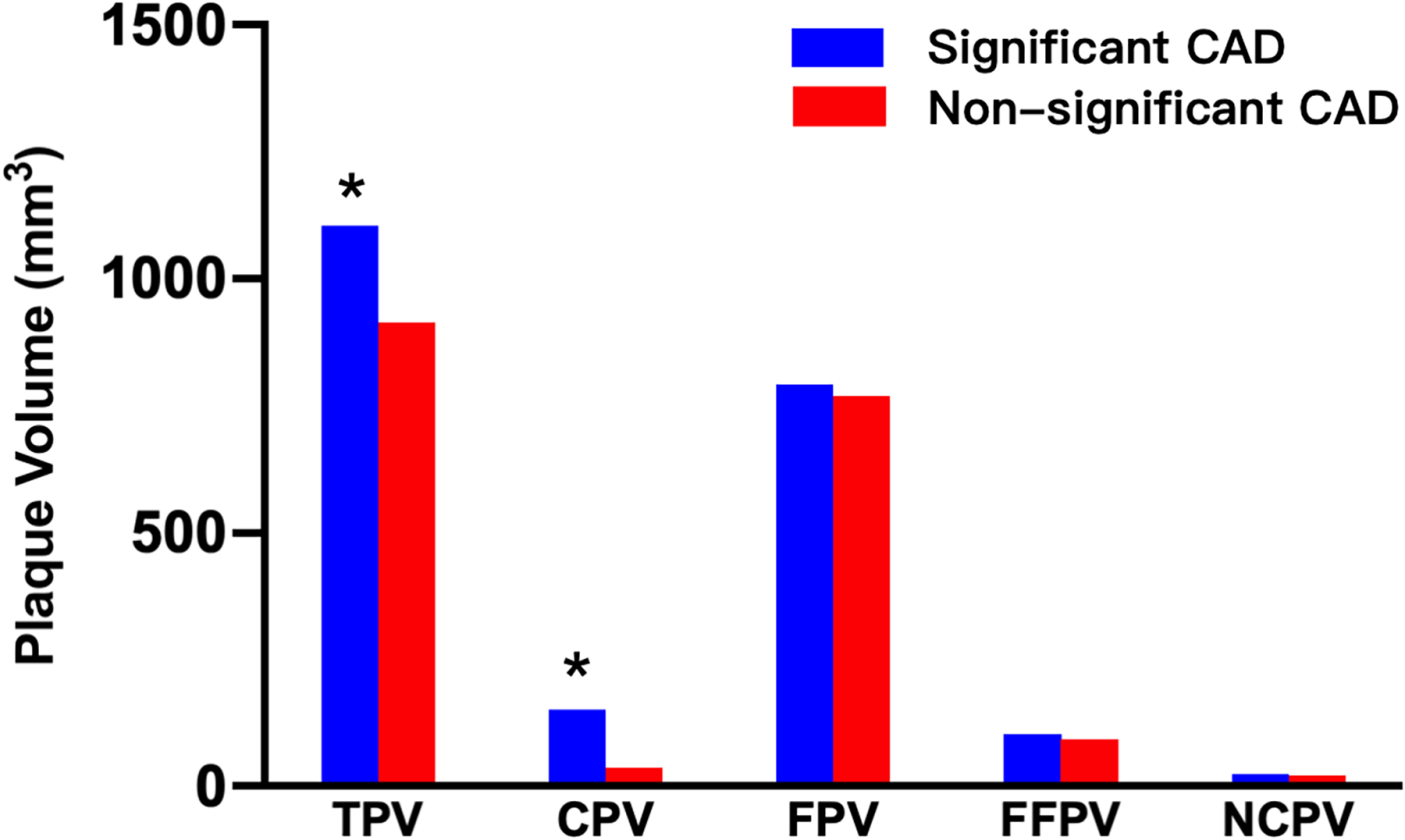
Whole-heart plaque volume by composition on CCTA for patients with significant and non-significant CAD. *p < 0.05 between groups. TPV, total plaque volume; CPV, calcified plaque volume; FPV, fibrous plaque volume; FFPV, fibro-fatty plaque volume; NCPV, necrotic core plaque volume.

Lesion measurements on CCTA showed that patients with significant CAD had larger maximal diameter stenosis (57.36% ± 9.56% vs. 30.67% ± 7.01%; *p* < 0.001), maximal area stenosis (79.63% ± 11.69% vs. 51.45% ± 10.16%; *p* < 0.001), maximal cross-sectional plaque burden (80.18% ± 10.31% vs. 55.56% ± 9.72%; *p* < 0.001), and mean plaque burden (34.99% ± 5.16% vs. 30.83% ± 5.38%; *p* < 0.001) than patients without significant CAD. Plaque volumetric measurements showed that total plaque volume (1,103.50 ± 285.51 mm^3^ vs. 913.18 ± 277.45 mm^3^; *p* < 0.001) and calcified plaque volume (149.77 ± 160.71 mm^3^ vs. 36.42 ± 26.86 mm^3^; *p* < 0.001) were much higher in the significant CAD group than in the non-significant CAD group. No significant differences were observed in fibrous plaque volume, fiber-fatty plaque volume, and necrotic core plaque volume between the two groups (all *p* > 0.05) (Table 2, Figure 4).

**Table 2:** CT measurements.

Parameters	Significant CAD	Non-significant CAD	*p*-value
	*N* = 49	*N* = 168	
Calcium scoring (Agatston score)	324.28 ± 389.02	39.16 ± 68.88	<0.001
Agatston score, *n* (%)			<0.001
1–100	22 (44.9)	154 (91.7)	
101–300	11 (22.4)	12 (7.1)	
>300	16 (32.7)	2 (1.2)	
Atherosclerotic feature			
Maximal			
Diameter stenosis (%)	57.36 ± 9.56	30.67 ± 7.01	<0.001
Area stenosis (%)	79.63 ± 11.69	51.45 ± 10.16	<0.001
Cross-sectional plaque burden (%)	80.18 ± 10.31	55.56 ± 9.72	<0.001
Mean plaque burden (%)	34.99 ± 5.16	30.83 ± 5.38	<0.001
Total plaque volume (mm^3^)	1,103.50 ± 285.51	913.18 ± 277.45	<0.001
Entire LCA	626.65 ± 150.88	523.05 ± 200.30	0.003
LM	61.34 ± 38.99	52.98 ± 27.99	0.155
LAD	361.56 ± 124.16	302.62 ± 107.68	0.005
LCX	185.39 ± 64.60	192.30 ± 89.21	0.654
RCA	486.55 ± 224.11	149.77 ± 160.71	0.007
Calcified plaque volume (mm^3^)	149.77 ± 160.71	36.42 ± 26.86	<0.001
Entire LCA	87.40 ± 74.59	26.18 ± 22.31	<0.001
LM	6.52 ± 14.93	2.47 ± 4.50	0.096
LAD	67.39 ± 63.60	19.43 ± 17.51	<0.001
LCX	13.49 ± 16.79	4.28 ± 6.40	0.001
RCA	62.36 ± 104.06	10.24 ± 12.82	0.003
Fibrous plaque volume (mm^3^)	790.16 ± 252.35	766.57 ± 149.6	0.609
Entire LCA	428.13 ± 139.91	434.57 ± 163.24	0.822
LM	49.07 ± 25.98	44.81 ± 23.48	0.359
LAD	234.55 ± 91.78	231.00 ± 99.25	0.842
LCX	151.79 ± 64.23	161.95 ± 82.39	0.474
RCA	362.03 ± 166.23	332.00 ± 161.84	0.316
Fibro-fatty plaque volume (mm^3^)	101.22 ± 23.11	92.24 ± 29.19	0.083
Entire LCA	64.65 ± 16.66	58.11 ± 22.04	0.091
LM	3.69 ± 3.42	3.50 ± 2.41	0.717
LAD	41.77 ± 13.13	36.81 ± 16.64	0.092
LCX	19.18 ± 6.27	17.80 ± 8.09	0.33
RCA	36.57 ± 11.01	34.13 ± 14.28	0.33
Necrotic core plaque volume (mm^3^)	23.47 ± 12.47	20.44 ± 11.69	0.175
Entire LCA	16.48 ± 10.01	13.94 ± 11.08	0.192
LM	0.80 ± 1.63	0.63 ± 1.03	0.48
LAD	11.51 ± 8.33	9.35 ± 10.01	0.226
LCX	4.17 ± 2.85	3.96 ± 3.25	0.724
RCA	6.99 ± 4.90	6.49 ± 5.81	0.632

LAD, left anterior descending; LCA, left coronary artery; LCX, left circumflex; LM, left main; RCA, right coronary artery.

**Table 3:** Independent predictors of significant CAD.

Variables	Univariate analysis			Multivariate analysis		
	OR	CI	*p*-value	OR	CI	*p*-value
Agatston score	1.01	1.006–1.014	<0.001	1.01	1.005–1.014	<0.001
Age	1.055	1.009–1.103	0.018	1.954	0.788–4.847	0.148
BMI	0.974	0.852–1.114	0.700			
Smoking	2.524	1.179–5.399	0.017	1.008	0.958–1.060	0.761
Hypertension	1.189	0.563–2.513	0.650			
Diabetes mellitus	1.125	0.491–2.578	0.781			
Dyslipidemia	1.521	0.802–2.886	0.199			
Total flight time	1.000	1.000–1.000	0.062			
Flight time/year	0.999	0.998–1.001	0.351			
Beta-blockers	1.761	0.424–7.315	0.436			
Calcium antagonists	0.841	0.298–2.370	0.743			
ACEI/ARB	1.033	0.390–2.733	0.949			
Hypoglycemic agents	1.106	0.442–2.768	0.829			
Statins	1.387	0.261–7.381	0.701			

ACEI, angiotensin-converting enzyme inhibitors; ARB, angiotensin II receptor blockers; BMI, body mass index; CI, confidence interval; OR, odds ratio.

### Independent predictor of significant CAD

Independent predictors of significant CAD were evaluated using logistic regression analysis. CACS [odds ratio (OR) 1.01, 95% confidence interval (CI) 1.005–1.014; *p* < 0.001] was the only independent risk factor of significant CAD; no other traditional cardiovascular risk factors, including age, smoking, hypertension, diabetes mellitus, dyslipidemia, and pre-existing medication regimens, showed significant associations. In addition, flight time, including total flight time and flight time/year, were not found to be independent risk factors of significant CAD (Table 3).

### Association between CACS and plaque volume

CACS is positively correlated with total plaque volume (*r* = 0.156; *p* = 0.027) and calcified plaque volume (*r* = 0.434; *p* < 0.001). No significant correlation of Agatston CACS with fibrous plaque volume (*r* = 0.012; *p* = 865), fibro-fatty plaque volume (*r* = 0.067; *p* = 0.341), and necrotic core plaque volume (*r* = 0.100; *p* = 0.154) were found.

### Receiver operating characteristic curve for CACS

**Figure 5:**
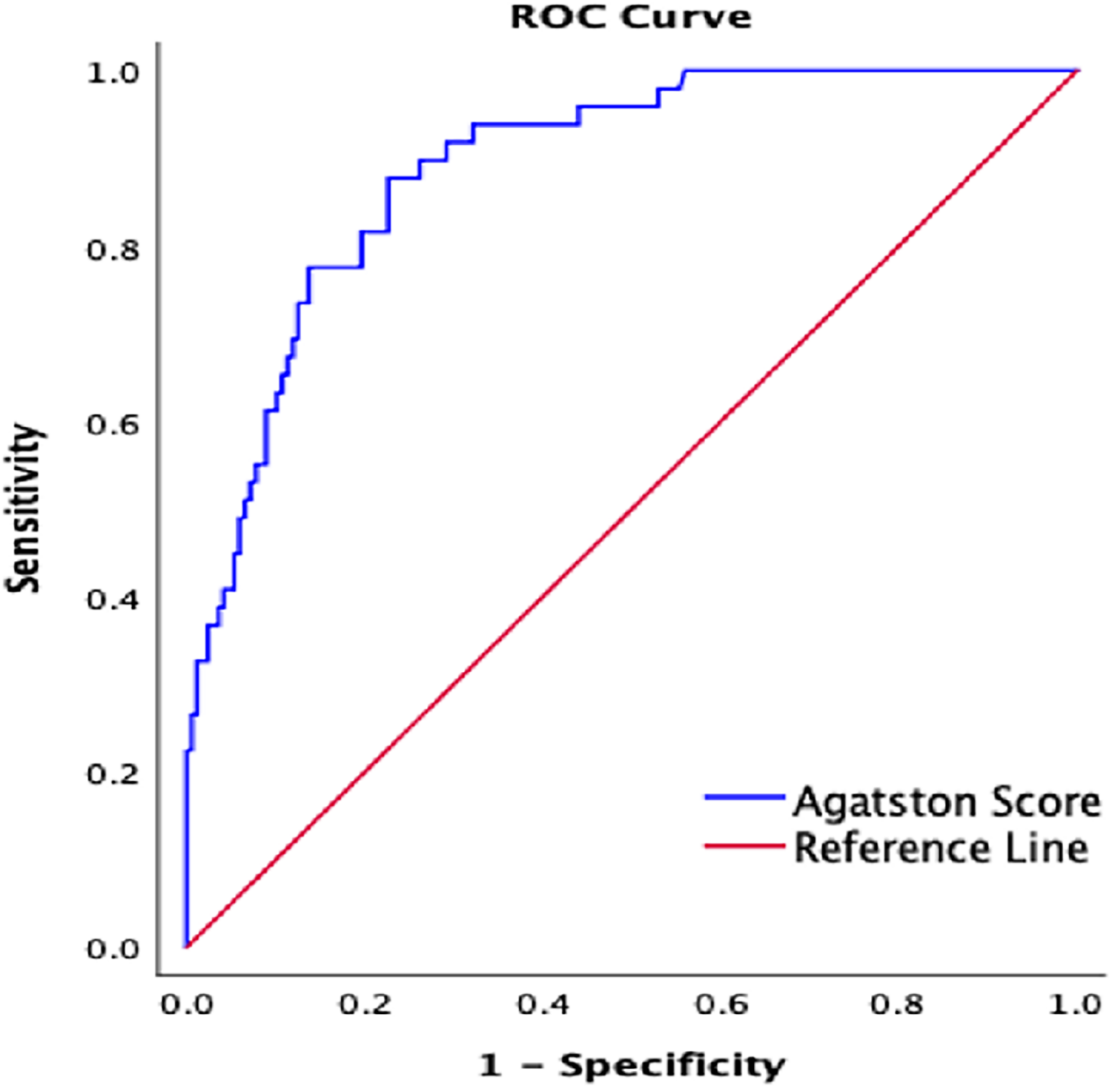
Receiver operator characteristic curve for significant CAD.

According to the receiver operating characteristic (ROC) curve analysis, the area under the ROC curve (AUC) for the Agatston CACS for diagnosing significant CAD in pilots was 0.891 (*p* < 0.001), indicating excellent discriminative ability. At the optimal cutoff value of 46, the sensitivity was 87.8% and the specificity was 77.4%. The positive predictive value was 75%, the negative predictive value was 83.9%, and the accuracy was 82.9%. These results suggested that CACS could accurately predict the severity of coronary stenosis in airline pilots (Figure 5).

## Discussion

To the best of our knowledge, this was a novel study that investigated the value of CACS based on NCCT for the assessment of significant CAD specializing in airline pilots in China. Our study demonstrated that CACS assessed by NCCT was associated with plaque burden, including total and calcified plaque volume, and could effectively predict CAD-RADS category 3 or higher. NCCT may serve as a promising and effective tool for early screening of significant CAD risk among airline pilots.

Maintaining optimal physical health for flight duties in pilots is of paramount importance as they are responsible for passenger safety and flight operations. Sudden in-flight incapacitation or even death among pilots is often caused by CVD, especially CAD, and it has been well-documented as one of the most serious threats to aviation safety (14, 15). Therefore, the early detection of significant CAD in pilots to avoid MI or sudden death is pivotal to prevent potentially catastrophic airplane accidents. Although annual physical examinations are routinely conducted to monitor heath conditions, identifying pilots at high risk of CAD remains challenging. NCCT offers a feasible approach for large-scale early risk stratification and CAD screening in the pilot population. CAC, a hallmark of atherosclerosis, can be quantified by NCCT in a simple, rapid, and reliable manner using the CACS (16–18). Previous studies have demonstrated a strong correlation between the degree of luminal stenosis and the extent of coronary calcification (18–20). Moradi et al. emphasized that CACS could predict CAD-RADS score, a robust and well-established diagnostic tool for assessing CAD severity (21)—a perspective consistent with our findings in the pilot population, which is critically important for aviation medicine and the implementation of pilot health surveillance programs. Furthermore, CACS has been extensively established as a strong prognostic predictor of major cardiovascular outcomes in asymptomatic individuals (8, 19, 22, 23).

Our study has demonstrated the predictive value of CACS for significant CAD defined by CAD-RADS score using CCTA, rather than invasive coronary angiography (ICA), which remains the gold standard for CAD assessment in clinical practice (24). CCTA is widely recognized as a robust, non-invasive, highly effective tool for evaluating the severity of coronary artery stenosis. Numerous studies have shown high concordance and comparable accuracy between CCTA and ICA in identifying patients with significant CAD (12, 25–27). However, the diagnostic accuracy of CCTA might be reduced in case of severe calcification or imaging artifacts, primarily due to limitations in imaging resolution (27, 28). These challenges could potentially be mitigated by the application of innovative photon-counting CT technology as its comparable imaging resolution to ICA reported (29, 30). Previous studies had documented the correlation between NCCT-CACS and plaque burden as well as plaque components by CTA (19, 20, 31), which aligns with the findings of our study. In addition, CCTA-derived plaque volumes, morphology, and composition have illustrated significant prognostic value for predicting cardiovascular events (32–34).

The use of medication, particularly statins, may influence the progression of atherosclerotic plaque. The PARADIGM study, which included 654 patients with available statin usage data and serial CCTA, demonstrated that CACS progression reflected the progression of total PVs and calcified PV in both the statin-naive group and statin-treated group. Nevertheless, in patients receiving statins, CACS was associated only with calcified PV progression, not with non-calcified PV progression (35). Similarly, Shi et al. reported that an increase of CACS in statins-treated patients with DM indicated the progression of compositional PVs (11). In our study, no significant difference in statin usage was observed between the significant and non-significant CAD groups. However, only a small proportion of the cohort had a history of statin therapy (4.1% in the significant CAD group and 3.0% in the non-significant CAD group), limiting the ability to comprehensively evaluate the effects of statin use on the current findings.

Given the limitations of statin therapy evaluation in our study, it is crucial to consider broader strategies for screening and managing CAD, especially in high-risk populations such as airline pilots. The management of CAD in this unique group requires a comprehensive approach, as highlighted by Davenport et al. (36). Specifically, they emphasized that aircrew with a history of myocardial infarction or revascularization necessitates rigorous risk stratification, regular monitoring, and tailored interventions. Consequently, an optimal strategy for CAD screening and management in airline pilots should integrate advanced diagnostic tools, personalized treatment plans, and collaborative decision-making among healthcare professionals. Achieving a balance between aviation safety and individual health outcomes is paramount, particularly considering the high-risk nature of their profession and the potentially catastrophic consequences of in-flight cardiac events.

Building on the utility of CACS in high-risk populations like airline pilots, our study has demonstrated its effectiveness as a predictor of significant CAD in high-stress occupations. This approach may also extend to other safety-sensitive or high-stress professions, such as firefighters, police officers, and healthcare workers in emergency rooms or intensive care units. For the general population, CACS could serve as a valuable tool for risk stratification, particularly in individuals with intermediate risk factors. However, its widespread adoption would require careful consideration of cost-effectiveness, potential for overdiagnosis, and ethical implications. Ultimately, the use of CACS should be guided by evidence-based guidelines and tailored to the specific needs of the population being screened, ensuring a balance between early detection and the avoidance of unnecessary interventions.

Our study leverages CACS derived from non-gated NCCT scans, initially performed for lung cancer screening, to predict significant CAD. This opportunistic approach enhances the diagnostic and preventive capabilities of medical imaging by extracting additional insights from existing scans. The idea of our study aligns well with very recent studies using artificial intelligence (AI) to predict the atherosclerotic burden or coronary event risk based on imaging modalities such as chest X-ray and lung CT scans (37–39). These developments in AI further underscore the clinical utility of opportunistic imaging interpretation, paving the way for more comprehensive and efficient diagnostic strategies.

### Limitations

Our study has some limitations. The definition of significant CAD was made using CCTA but not ICA; therefore, the possibility of false-positive and false-negative CCTA findings might exist despite the performance of CCTA by experienced experts. The CACS was assessed using non-gated NCCT with a reconstructed slice thickness of 1 mm and a slice interval of 0.7 mm, as opposed to the standard ECG-gated technique with a 3 mm slice thickness (40), potentially introducing systematic bias. The absence of validation between these two methods represents a significant limitation, suggesting that the current methodology needs verification before it can be used in external cohorts. The sample size was relatively small and women were excluded from the study. In addition, high-risk plaque (HRP) has been shown to independently increase the risk of MI, irrespective of calcium score, obstructive disease, gender, or cardiovascular risk factors (41). Although the absence of CAC reduces the likelihood of significant CAD, it does not completely rule out its occurrence (42). Further studies are needed to investigate the relationship between CACS and HRP, as well as to identify additional screening methods to effectively evaluate CAD in pilots without detectable CAC.

## Conclusions

CACS assessed by NCCT was significantly associated with CAD-RADS 3 or higher, as confirmed by CCTA, indicating that it may serve as a robust predictor for diagnosing significant CAD among airline pilots. Therefore, CACS based on NCCT holds potential as a promising and effective tool for early screening of significant CAD risk in the airline pilot population.

## Data Availability

The raw data supporting the conclusions of this article will be made available by the authors, without undue reservation.

## References

[1] Feigin VL Brainin M Norrving B Martins S Sacco RL Hacke W World Stroke Organization (WSO): global stroke fact sheet 2022. Int J Stroke. (2022) 17:18–29. 10.1177/17474930211065917 34986727

[2] GBD 2019 Stroke C. Global, regional, and national burden of stroke and its risk factors, 1990–2019: a systematic analysis for the global burden of disease study 2019. Lancet Neurol. (2021) 20:795–820. 10.1016/S1474-4422(21)00252-0 34487721 PMC8443449

[3] Sutton NR Banerjee S Cooper MM Arbab-Zadeh A Kim J Arain MA Coronary artery disease evaluation and management considerations for high risk occupations: commercial vehicle drivers and pilots. Circ Cardiovasc Interv. (2021) 14:e009950. 10.1161/CIRCINTERVENTIONS.120.009950 34092098

[4] Choi Y Kim K. Effects of physical examination and diet consultation on serum cholesterol and health-behavior in the Korean pilots employed in commercial airline. Ind Health. (2013) 51:603–11. 10.2486/indhealth.2012-0027 24131872 PMC4202750

[5] Cury RC Leipsic J Abbara S Achenbach S Berman D Bittencourt M CAD-RADS™ 2.0–2022 coronary artery disease - reporting and data system.: an expert consensus document of the Society of Cardiovascular Computed Tomography (SCCT), the American College of Cardiology (ACC), the American College of Radiology (ACR) and the North America Society of Cardiovascular Imaging (NASCI). J Am Coll Radiol. (2022) 19(11):1185–212. 10.1016/j.jacr.2022.09.012 36436841

[6] Rumberger J Simons D Fitzpatrick L Sheedy P Schwartz R. Coronary artery calcium area by electron-beam computed tomography and coronary atherosclerotic plaque area. A histopathologic correlative study. Circulation. (1995) 92:2157–62. 10.1161/01.CIR.92.8.2157 7554196

[7] Vrints C Andreotti F Koskinas KC Rossello X Adamo M Ainslie J 2024 ESC guidelines for the management of chronic coronary syndromes. Eur Heart J. (2024) 45:3415–537. 10.1093/eurheartj/ehae177 39210710

[8] Schmermund A Möhlenkamp S Stang A Grönemeyer D Seibel R Hirche H Assessment of clinically silent atherosclerotic disease and established and novel risk factors for predicting myocardial infarction and cardiac death in healthy middle-aged subjects: rationale and design of the Heinz Nixdorf RECALL study. Risk factors, evaluation of coronary calcium and lifestyle. Am Heart J. (2002) 144:212–8. 10.1067/mhj.2002.123579 12177636

[9] Emfietzoglou M Mavrogiannis MC Samaras A Rampidis GP Giannakoulas G Kampaktsis PN. The role of cardiac computed tomography in predicting adverse coronary events. Front Cardiovasc Med. (2022) 9:920119. 10.3389/fcvm.2022.920119 35911522 PMC9334665

[10] McCollough C Ulzheimer S Halliburton S Shanneik K White R Kalender W. Coronary artery calcium: a multi-institutional, multimanufacturer international standard for quantification at cardiac CT. Radiology. (2007) 243:527–38. 10.1148/radiol.2432050808 17456875

[11] Shi R Gao Y Shen L-L Shi K Wang J Jiang L The effect of LDL-C status on the association between increased coronary artery calcium score and compositional plaque volume progression in statins-treated diabetic patients: evaluated using serial coronary CTAs. Cardiovasc Diabetol. (2022) 21:121. 10.1186/s12933-022-01556-y 35773708 PMC9248151

[12] Cucoranu D Pop M Niculescu R Kosovski I Toganel R Licu R Correlation between coronary artery disease and non-alcoholic fatty liver disease using computed tomography coronary calcium scans. Curr Health Sci. (2023) 49:244–50. 10.12865/CHSJ.49.02.244 PMC10541065 37779834

[13] Narula J Chandrashekhar Y Ahmadi A Abbara S Berman DS Blankstein R SCCT 2021 expert consensus document on coronary computed tomographic angiography: a report of the society of cardiovascular computed tomography. J Cardiovasc Comput Tomogr. (2021) 15:192–217. 10.1016/j.jcct.2020.11.001 33303384 PMC8713482

[14] Taneja N Wiegmann D. Prevalence of cardiovascular abnormalities in pilots involved in fatal general aviation airplane accidents. Aviat Space Environ Med. (2002) 73:1025–30. 12398267

[15] Whitton R. Medical disqualification in USAF pilots and navigators. Aviat Space Environ Med. (1984) 55:332–6. 6732686

[16] Genders TSS Pugliese F Mollet NR Meijboom WB Weustink AC van Mieghem CAG Incremental value of the CT coronary calcium score for the prediction of coronary artery disease. Eur Radiol. (2010) 20:2331–40. 10.1007/s00330-010-1802-y 20559838 PMC2940023

[17] Thilo C Gebregziabher M Mayer FB Zwerner PL Costello P Schoepf UJ. Correlation of regional distribution and morphological pattern of calcification at CT coronary artery calcium scoring with non-calcified plaque formation and stenosis. Eur Radiol. (2009) 20:855–61. 10.1007/s00330-009-1630-0 19862532

[18] Becker C Knez A Ohnesorge B Schoepf U Flohr T Bruening R Visualization and quantification of coronary calcifications with electron beam and spiral computed tomography. Eur Radiol. (2000) 10:629–35. 10.1007/s003300050975 10795546

[19] Bild DE. Multi-ethnic study of atherosclerosis: objectives and design. Am J Epidemiol. (2002) 156:871–81. 10.1093/aje/kwf113 12397006

[20] Kaur M Rahimi R Razali F Mohd Noor N Omar E Abdul Manaf Z Association of coronary artery calcium score with calcification and degree of stenosis: an autopsy study. Malays J Pathol. (2019) 41:177–83. 31427553

[21] Moradi M Rafiei E Rasti S Haghbin H. Coronary artery calcification—does it predict the CAD-RADS category? Emerg Radiol. (2022) 29:969–77. 10.1007/s10140-022-02082-w 35922681 PMC9362466

[22] Hoffmann U Massaro JM Fox CS Manders E O'Donnell CJ. Defining normal distributions of coronary artery calcium in women and men (from the Framingham heart study). Am J Cardiol. (2008) 102:1136–1141.e1. 10.1016/j.amjcard.2008.06.038 18940279 PMC3065378

[23] Shlomai G Shemesh J Segev S Koren-Morag N Grossman E. The multi-ethnic study of atherosclerosis-calcium score improves statin treatment allocation in asymptomatic adults. Front Cardiovasc Med. (2022) 9:855390. 10.3389/fcvm.2022.855390 35911540 PMC9334900

[24] Lim MJ White CJ. Coronary angiography is the gold standard for patients with significant left ventricular dysfunction. Progress in cardiovascular diseases. Prog Cardiovasc Dis. (2013) 55:504–8. 10.1016/j.pcad.2013.01.003 23518380

[25] Vattay B Borzsák S Boussoussou M Vecsey-Nagy M Jermendy ÁL Suhai FI Association between coronary plaque volume and myocardial ischemia detected by dynamic perfusion CT imaging. Front Cardiovasc Med. (2022) 9:974805. 10.3389/fcvm.2022.974805 36158821 PMC9498180

[26] Howden N Branch K Douglas P Gray M Budoff M Dewey M Computed tomographic angiography measures of coronary plaque in clinical trials: opportunities and considerations to accelerate drug translation. Front Cardiovasc Med. (2024) 11:1359500. 10.3389/fcvm.2024.1359500 38500753 PMC10945423

[27] Tekinhatun M Akbudak İ Özbek M Turmak M. Comparison of coronary CT angiography and invasive coronary angiography results. Ir J Med Sci. (2024) 193(5):2239–48. 10.1007/s11845-024-03745-y 38965116 PMC11450059

[28] Liu L Yang W Nagahara Y Li Y Lamooki SR Muramatsu T The impact of image resolution on computation of fractional flow reserve: coronary computed tomography angiography versus 3-dimensional quantitative coronary angiography. Int J Cardiovasc Imaging. (2016) 32(3):513–23. 10.1007/s10554-015-0797-5 26507326

[29] Schuijf JD Lima JAC Boedeker KL Takagi H Tanaka R Yoshioka K CT imaging with ultra-high-resolution: opportunities for cardiovascular imaging in clinical practice. J Cardiovasc Comput Tomogr. (2022) 16(5):388–96. 10.1016/j.jcct.2022.02.003 35210183

[30] Mergen V Eberhard M Manka R Euler A Alkadhi H. First in-human quantitative plaque characterization with ultra-high resolution coronary photon-counting CT angiography. Front Cardiovasc Med. (2022) 9:981012. 10.3389/fcvm.2022.981012 36148053 PMC9485480

[31] Tiansuwan N Sasiprapha T Jongjirasiri S Unwanatham N Thakkinstian A Laothamatas J Utility of coronary artery calcium in refining 10-year ASCVD risk prediction using a Thai CV risk score. Front Cardiovasc Med. (2023) 10:1264640. 10.3389/fcvm.2023.1264640 38028497 PMC10652894

[32] Andreini D Magnoni M Conte E Masson S Mushtaq S Berti S Coronary plaque features on CTA can identify patients at increased risk of cardiovascular events. JACC Cardiovasc Imaging. (2020) 13:1704–17. 10.1016/j.jcmg.2019.06.019 31422137

[33] Versteylen MO Kietselaer BL Dagnelie PC Joosen IA Dedic A Raaijmakers, RH Additive value of semiautomated quantification of coronary artery disease using cardiac computed tomographic angiography to predict future acute coronary syndrome. J Am Coll Cardiol. (2013) 61:2296–305. 10.1016/j.jacc.2013.02.065 23562925

[34] Chang H-J Lin FY Lee S-E Andreini D Bax J Cademartiri F Coronary atherosclerotic precursors of acute coronary syndromes. J Am Coll Cardiol. (2018) 71:2511–22. 10.1016/j.jacc.2018.02.079 29852975 PMC6020028

[35] Lee S-E Sung JM Andreini D Budoff MJ Cademartiri F Chinnaiyan K Differential association between the progression of coronary artery calcium score and coronary plaque volume progression according to statins: the Progression of AtheRosclerotic PlAque DetermIned by Computed TomoGraphic Angiography Imaging (PARADIGM) study. Eur Heart J Cardiovasc Imaging. (2019) 20:1307–14. 10.1093/ehjci/jez022 30789215 PMC6806249

[36] Davenport ED Syburra T Gray G Rienks R Bron D Manen O Management of established coronary artery disease in aircrew with previous myocardial infarction or revascularisation. Heart. (2019) 105(Suppl 1):s31–7. 10.1136/heartjnl-2018-313055 30425084 PMC6256305

[37] Abdelrahman K Shiyovich A Huck DM Berman AN Weber B Gupta S Artificial intelligence in coronary artery calcium scoring detection and quantification. Diagnostics (Basel). (2024) 14(2):125. 10.3390/diagnostics14020125 38248002 PMC10814920

[38] Acosta JN Falcone GJ Rajpurkar P Topol EJ. Multimodal biomedical AI. Nat Med. (2022) 28(9):1773–84. 10.1038/s41591-022-01981-2 36109635

[39] Topol EJ. AI-enabled opportunistic medical scan interpretation. Lancet. (2024) 403(10439):1842. 10.1016/S0140-6736(24)00924-3 38735291

[40] Shin JM Kim TH Kim JY Park CH. Coronary artery calcium scoring on non-gated, non-contrast chest computed tomography (CT) using wide-detector, high-pitch and fast gantry rotation: comparison with dedicated calcium scoring CT. J Thorac Dis. (2020) 12(10):5783–93. 10.21037/jtd-20-1371 33209410 PMC7656362

[41] Williams MC Kwiecinski J Doris M McElhinney P D’Souza MS Cadet S Sex-specific computed tomography coronary plaque characterization and risk of myocardial infarction. JACC Cardiovasc Imaging. (2021) 14(9):1804–14. 10.1016/j.jcmg.2021.03.004 33865779 PMC8435010

[42] Villines TC Hulten EA Shaw LJ Goyal M Dunning A Achenbach S Prevalence and severity of coronary artery disease and adverse events among symptomatic patients with coronary artery calcification scores of zero undergoing coronary computed tomography angiography. J Am Coll Cardiol 2011;58:2533–40. 10.1016/j.jacc.2011.10.851 22079127

